# Spontaneous Improvement of Hypogonadotropic Hypogonadism in a Patient with *PCSK1* and *HS6ST1* Mutations: A Case Report

**DOI:** 10.3390/life15071151

**Published:** 2025-07-21

**Authors:** Alanna Asgeirsson, Eujean Park, Vinicius Seidel, Mathew Shedd, Matheni Sathananthan, Tania Arous, Kevin Codorniz, Silvana Giannelli, Justin Do, Wyut Yi Thin, Arsenije Jelovac, Scott Lee

**Affiliations:** 1 Department of Internal Medicine, Loma Linda University Health Consortium, Loma Linda, CA 92354, USA; aasgeirsson@llu.edu (A.A.); vseidel@llu.edu (V.S.); mshedd@llu.edu (M.S.); 2 Division of Endocrinology, Loma Linda University Health Consortium, Loma Linda, CA 92354, USA; eujeanpark@llu.edu (E.P.); msathananthan@llu.edu (M.S.); tarous@llu.edu (T.A.); sgiannelli@llu.edu (S.G.); jdo@llu.edu (J.D.); wthin@llu.edu (W.Y.T.); 3 Providence Little Company of Mary Medical Center Torrance, Torrance, CA 4101, USA

**Keywords:** spontaneous hypogonadotropic hypogonadism reversal, *PCSK1* mutation, *HS6ST1* mutation, GnRH neurons

## Abstract

Kallmann syndrome (KS) is a form of hypogonadotropic hypogonadism (HH) characterized by gonadotropin-releasing hormone (GnRH) deficiency and anosmia due to defective neuronal migration. While traditionally considered irreversible, cases of spontaneous improvement of HH have been reported, suggesting residual GnRH neuronal function in some individuals. We present a case of a 29-year-old man with KS who exhibited spontaneous recovery of endogenous testosterone production following the cessation of long-term androgen therapy without the use of alternative hormonal agents. After ceasing testosterone therapy for several months, the patient’s total testosterone levels normalized (407–424 ng/dL), accompanied by increased secondary sexual characteristics, stable gonadotropin levels, and normal testicular volume. Persistent anosmia was noted, suggesting that restoration of reproductive endocrine function can occur independently of olfactory recovery. Genetic testing identified heterozygous mutations in *PCSK1* and *HS6ST1*, genes implicated in GnRH regulation and KS pathogenesis. This case highlights the potential role of genetic variation in spontaneous HH improvement and underscores the need for individualized management strategies, including periodic reassessment of gonadal function and fertility potential. Further research is needed to elucidate the mechanisms driving spontaneous HH improvement, identify predictive biomarkers of reversibility, and explore therapeutic strategies that may promote endogenous GnRH activity in select patients with KS.

## 1. Introduction

Hypogonadotropic hypogonadism (HH) is a condition caused by THE insufficient secretion of gonadotropin-releasing hormone (GnRH). Kallmann syndrome (KS) is a form of HH, distinguished by the presence of anosmia or hyposmia. Although its pathophysiology is not fully understood, it is believed to involve olfactory and GnRH neurons failing to migrate properly during embryonic development, leading to its characteristic anosmia and a spectrum of other clinical features, including absent or incomplete sexual maturation and possible infertility [1,2]. Prevalence remains relatively unclear, but it is estimated to be 1 out of 80,000 in men, although it may be underdiagnosed due to anosmia often going unnoticed or unreported. The prevalence in women is thought to be approximately five times lower than that of men, but this may be an underestimation due to women often presenting with more mild symptoms [1].

While traditionally considered a lifelong condition, there have been documented cases of spontaneous normalization of the hypothalamic-pituitary–gonadal axis, allowing for the resumption of endogenous testosterone production and the development of secondary sexual characteristics [2]. Several mechanisms have been proposed, including the possibility that exogenous androgens may promote the maturation or reactivation of GnRH neurons in patients with partially preserved neuronal architecture [3]. However, the etiology of such reversals remains poorly understood. For example, while it is known that *ANOS1* and *FGFR1* genetic mutations play a role in the development of KS itself, some individuals with *FGFR1* mutations (and at least one instance of *ANOS1* mutation [4]) have documented “reversal of KS”, possibly due to retained residual GnRH neuronal function [2,5]. This may imply that the genes responsible for KS can also influence the likelihood and extent of spontaneous reversal, depending on the nature and severity of the mutations.

This case report examines an unusual case of spontaneous HH improvement in a patient with heterozygous mutations in the *PCSK1* and *HS6ST1* genes. These genes are less frequently implicated in KS compared to more established variants like *FGFR1* and *ANOS1*, making this case particularly valuable in exploring underrecognized molecular contributors to GnRH dysfunction and variability in clinical course. Furthermore, we highlight the clinical importance of periodic reassessment in KS patients, as such re-evaluation may reveal spontaneous improvement in hypothalamic function and alter the therapeutic trajectory.

## 2. Detailed Case Description

A 25-year-old man presented to our endocrinology clinic with decreased libido, gynecomastia (greater on the left), fatigue, mood swings, and muscular atrophy despite regular exercise and high protein intake. He had scant body and facial hair growth, though pubic and axillary hair were present. The patient recalled going through puberty later than his peers, occurring around age 16. He reported anosmia since childhood. He also described his testes as small but denied any history of mumps or testicular injuries. There was no family history of hypogonadism, and his three brothers were able to grow facial hair. He denied the use of testosterone, supplements, or medications known to affect the hypothalamic-pituitary-gonadal axis. A few months prior to presenting to the clinic, he had purchased a home fingerstick testosterone test, which was reportedly low.

On physical examination, gynecomastia was noted (left greater than right) without tenderness or nipple discharge. Central obesity was noted without abdominal striae. No testicular examination was documented at that time. The musculoskeletal, neurological, and psychiatric exams were unremarkable.

Laboratory tests ordered by his primary care physician showed a low serum total morning testosterone level of 77 ng/dL and free morning testosterone level of 0.92 ng/dL, with inappropriately normal luteinizing hormone (LH) of 2.0 mIU/mL and follicle-stimulating hormone (FSH) of 2.3 mIU/mL. Genetic testing at the time revealed that he is heterozygous in the *PCSK1* gene for a sequence variant designated c.661A>G, which is predicted to result in the amino acid substitution p.Asn221Asp. The report also showed that while he is heterozygous in the *HS6ST1* gene for a sequence variant designated c.1125C>T, it is not predicted to result in an amino acid substitution. However, this variant may result in the creation of a novel splice donor site within exon 2. MRI of the brain with pituitary protocol was unremarkable and showed a normal optic chiasm and pituitary gland. We have no specific comments regarding the presence or absence of olfactory structures, although they did not appear to be visible. The patient was diagnosed with KS and initiated on testosterone and clomiphene therapy. However, he was lost to follow-up.

Four years later, the patient, now aged 29, returned to re-establish care, having run out of testosterone injections for approximately 2 months without the use of any other androgen-containing products. He reported only a brief use of clomiphene therapy 3 years prior, and he had been utilizing testosterone injections for approximately 4 years before running out 2 months prior to returning to the clinic. Laboratory tests at clinic visits (2 months, 4 months, and 1 year and 3 months off therapy) showed increased and stable levels of total and free testosterone, LH, and FSH—all within the normal reference ranges, as shown in Table 1. The patient reported increased facial and body hair growth, deepening of his voice, and improved muscle mass compared to the past, even while on testosterone therapy. He denied decreased libido, erectile dysfunction, or low energy levels, although anosmia persisted. However, he reported persistent gynecomastia associated with discomfort.

The genital exam showed no gross abnormalities, no inguinal hernias bilaterally, a right testicular volume of approximately 18 cc, and a left testicular volume of approximately 20 cc. The patient was amenable to referral for fertility evaluation and semen analysis. The current plan includes monitoring testosterone levels every 3 months and plastic surgery referral for gynecomastia.

## 3. Discussion

KS is a genetically heterogeneous disease, with over 34 KS-linked genetic variants identified [6]. Our patient’s genetic testing revealed a heterozygous *PCSK1* mutation. The *PCSK1* gene encodes a precursor to proprotein convertase 1/3, which is involved in processing numerous prohormones, including pro-gonadotropin-releasing hormone (pro-GnRH). *PCSK1* gene mutation has been linked to various endocrine disorders, including KS, metabolic disturbances, increased appetite, insulin dysregulation, and potential predispositions to obesity [7,8]. This patient’s elevated body mass index of 31 kg/m^2^ and family history of obesity support this connection, suggesting *PCSK1* may play a role in his phenotype.

The patient also has a heterozygous mutation in the *HS6ST1* gene, which is another gene implicated in KS and spontaneous improvement of HH [9]. While the patient has a variant of uncertain significance, the mutation can result in a novel splice site according to the genetic report. One study revealed that disruption of *HS6ST1* signaling altered genes in the same biological processes as seen in a knockdown of *FGFR1*, one of the known genetic variants in KS. The study suggests that disruption of both signaling pathways can potentially impact the GnRH neuron signaling pathway by disrupting gene transcription via dysregulation of the transcription factor SOX9/SOX10 and the chromatin regulator CHD7, which are also associated with KS [6]. This genetic data also raises the possibility that genetic profiling may someday aid in predicting the likelihood of spontaneous improvement. Still, larger genotype-phenotype correlation studies would be needed before considering routine clinical use.

While mutations in *FGFR1* and *ANOS1* have been widely studied in HH, mutations in *PCSK1* and *HS6ST1* may reflect a distinct subset of patients with partially preserved GnRH function. This opens the possibility that certain mutations, particularly heterozygous or splice-site variants, confer a milder phenotype with latent potential for neuronal recovery. These more subtle disruptions might preserve downstream signaling cascades under certain physiologic conditions.

It should also be noted that genetic testing in our patient did not reveal mutations in other known HH-associated genes, including prokineticin receptor 2 (*PROKR2*). *PROK2R* mutations such as the Val274Asp variant have been associated with reversible forms of HH and Kallmann syndrome [10]. The absence of such mutations in this case highlights the possibility that spontaneous improvement may occur independently of known pathogenic variants, underscoring the potential role of unidentified or modifying genetic factors.

Pulsatile GnRH or gonadotropin therapy promotes puberty and fertility, whereas androgen therapy induces virilization in males. Patients with KS typically require lifelong testosterone replacement therapy to maintain male secondary sexual characteristics [2]. Reported cases of spontaneous HH improvement suggest that a small subset of KS patients may experience normalized testosterone levels and partial restoration of fertility upon cessation of hormone therapy [5]. Two small studies including HH and KS patients reported spontaneous improvement in about 10% of patients, and another study predicted a lifetime incidence of spontaneous improvement in 22% of patients [2,11,12]. A recent multicenter cross-sectional study identified a testicular volume greater than 4 mL as a potential predictor of reversal [13]. Testicular growth, a biomarker of gonadotropin secretion, may serve as a key indicator of HH improvement, particularly in patients with previously low testicular volume [2,12]. Although the mechanism behind the spontaneous improvement of KS is unclear, it may involve exogenous androgen therapy and its effects on the plasticity of GnRH-producing neurons in adulthood [2,12]. Additionally, persistent anosmia has been observed in other cases of KS improvement, such as our own, suggesting that GnRH neurons do not necessarily require an intact olfactory system to regain function [12]. However, it is important to note that relapse after spontaneous improvement may occur and should be monitored [9].

In clinical practice, it remains unclear when to consider a trial pause in exogenous hormone replacement therapy to assess for improved endogenous hormone production. A major barrier to identifying spontaneous HH improvement is the lack of structured reassessment protocols. Many patients continue lifelong hormone therapy without periodic evaluation for endogenous recovery. Provider unfamiliarity with spontaneous HH improvement further contributes to under-recognition. Based on current literature, a reasonable approach may be to consider a trial off therapy after at least 12–24 months of stable hormonal replacement and testicular maturation, especially in patients with atypical genotypes or clinical features suggestive of residual function [13]. Additionally, patient education at the time of diagnosis can set the expectation that spontaneous improvement, though rare, is possible and worth re-checking. Close biochemical and clinical monitoring during the pause is advised, including evaluation of testicular volume, LH and FSH levels, and testosterone levels. Symptoms of androgen deficiency, such as fatigue, decreased libido, and mood disturbances, may emerge within months in patients without sustained endogenous production, necessitating prompt re-initiation of therapy [12].

Fertility remains a key consideration in these cases, especially in younger patients. Our patient reported that he had not considered having children because he did not think it was an option due to his condition. Fertility evaluation should be offered to patients experiencing spontaneous KS improvement, as they often achieve normalized sperm density (ranging between 8 × 10^6^/mL and 58 × 10^6^/mL) and the ability to conceive [3]. One case report documented a patient with an *ANOS1* mutation who achieved normal serum testosterone levels, testicular enlargement, and successful conception after discontinuation of testosterone therapy [4]. The timeline of sperm recovery in comparison to other signs of HH reversal, such as increased testosterone levels, remains relatively unknown because sperm concentration is often not tested until other features of HH reversal become evident. Given these findings, conducting thorough fertility evaluations in patients experiencing spontaneous KS improvement can provide valuable information for patients who are interested in conceiving. Given the long-standing assumption of infertility in HH, patients and families should be counseled that spontaneous reversal may restore fertility, and that paternity is indeed possible in these rare cases.

In addition to genetic and clinical factors, it is important to consider alternative or contributing causes of reversible HH in this patient. One potential etiology includes autoimmune hypophysitis or hypothalamitis, which can present with transient HH. Although our patient’s MRI did not reveal abnormalities, subtle autoimmune inflammation may not always be radiographically evident. Testing for antipituitary antibodies may help exclude autoimmune-mediated HH and would have strengthened the diagnostic workup in this case [14]. Future cases of suspected reversible HH should consider such testing, especially when clinical or imaging findings are inconclusive. 

## 4. Conclusions

This report presents a case of spontaneous improvement of HH, marked by restoration of endogenous testosterone production after discontinuation of exogenous therapy. Genetic testing revealed potential contributions from the *PCSK1* and *HS6ST1* genes, suggesting that these genes may play a role in KS pathogenesis and clinical variability. This case emphasizes the importance of individualized management in KS and the need for patient education regarding the possibility of spontaneous improvement at the time of diagnosis. Educating patients at diagnosis about the potential, albeit rare, for spontaneous improvement may improve adherence to follow-up and provide psychological benefits. For many, the belief that fertility is permanently lost may deter them from pursuing future evaluations or family planning. Clinicians may consider periodic trials of testosterone therapy in stable KS patients, particularly those with milder phenotypes or atypical genotypes, under close supervision to assess for the restoration of endogenous sex hormone production. If recovery is confirmed, patients should be offered fertility testing. However, such monitoring may be inconsistent due to limited provider familiarity with HH reversibility. Creating guidelines for re-evaluation could facilitate earlier identification of endogenous recovery and reduce unnecessary lifelong hormonal therapy. Further research is essential to clarify the mechanisms underlying KS variability and reversibility, evaluate whether genetic testing can serve as a prognostic tool to identify those most likely to experience sustained reversal, and optimize management for these patients.

## Figures and Tables

**Table 1:** Summary of key laboratory findings with reference ranges.

	At Diagnosis (Age 25)	2 Months off Therapy * (Age 29)	4 Months off Therapy * (Age 29)	1 Year and 3 Months off Therapy (Age 30)
Total Testosterone (ng/dL)	77 (240–950)	424 (250–1100)	407 (250–1100)	448 (240–950)
Free Testosterone (ng/dL)	0.92 (5.25–20.7)	6.19 (3.5–15.5) **	5.89 (3.5–15.5) **	11.7 (5.05–19.8)
LH (mIU/mL)	2.0 (0.9–10.6)	2.1 (1.5–9.3)	2.3 (1.5–9.3)	4.7 (0.9–10.6)
FSH (mIU/mL)	2.3 (1.5–12.4)	3.8 (1.4–12.8)	3.7 (1.4–12.8)	3.7 (1.5–12.4)

* Differences in reference ranges reflect testing at different laboratories at various timepoints. ** Free testosterone labs collected at 2 months and 4 months off therapy were originally measured in pg/mL. These measurements were converted for the table (1 ng/dL = 10 pg/mL).

## Data Availability

The data presented in this study are available on request from the corresponding author due to privacy restrictions.

## Abbreviations

The following abbreviations are used in this manuscript:

*ANOS1*	Anosmin-1
CHD7	Chromodomain helicase DNA binding protein 7
*FGFR1*	Fibroblast growth factor receptor 1
FSH	Follicle-stimulating hormone
GnRH	Gonadotropin-releasing hormone
HH	Hypogonadotropic hypogonadism
*HS6ST1*	Heparan sulfate 6-O-sulfotransferase 1
KS	Kallmann syndrome
LH	Luteinizing hormone
MRI	Magnetic resonance imaging
*PCSK1*	Proprotein convertase subtilisin/kexin type 1
*PROKR2*	Prokineticin receptor 2
SOX9/10	SRY-box transcription factors 9 and 10
